# X-linked intellectual disability gene *CASK* regulates postnatal brain growth in a non-cell autonomous manner

**DOI:** 10.1186/s40478-016-0295-6

**Published:** 2016-03-31

**Authors:** Sarika Srivastava, Ryan McMillan, Jeffery Willis, Helen Clark, Vrushali Chavan, Chen Liang, Haiyan Zhang, Matthew Hulver, Konark Mukherjee

**Affiliations:** Virginia Tech Carilion Research Institute, Roanoke, VA 24016 USA; Department of Biological Sciences, Virginia Tech, Blacksburg, VA 24061 USA; Metabolic Phenotyping Core at Virginia Tech, Blacksburg, VA 24061 USA; Department of Human Nutrition, Foods, and Exercise, Virginia Tech, Blacksburg, VA 24061 USA

**Keywords:** CASK, MAGUK, Cerebellar hypoplasia, Non-cell autonomous, X-linked intellectual disability, Metabolism

## Abstract

The phenotypic spectrum among girls with heterozygous mutations in the X-linked intellectual disability (XLID) gene *CASK* (calcium/calmodulin-dependent serine protein kinase) includes postnatal microcephaly, ponto-cerebellar hypoplasia, seizures, optic nerve hypoplasia, growth retardation and hypotonia. Although *CASK* knockout mice were previously reported to exhibit perinatal lethality and a 3-fold increased apoptotic rate in the brain, *CASK* deletion was not found to affect neuronal physiology and their electrical properties. The pathogenesis of *CASK* associated disorders and the potential function of *CASK* therefore remains unknown. Here, using Cre-LoxP mediated gene excision experiments; we demonstrate that deleting *CASK* specifically from mouse cerebellar neurons does not alter the cerebellar architecture or function. We demonstrate that the neuron-specific deletion of *CASK* in mice does not cause perinatal lethality but induces severe recurrent epileptic seizures and growth retardation before the onset of adulthood. Furthermore, we demonstrate that although neuron-specific haploinsufficiency of *CASK* is inconsequential, the *CASK* mutation associated human phenotypes are replicated with high fidelity in *CASK* heterozygous knockout female mice (*CASK*^(+/-)^). These data suggest that *CASK*-related phenotypes are not purely neuronal in origin. Surprisingly, the observed microcephaly in *CASK*^(+/-)^ animals is not associated with a specific loss of *CASK* null brain cells indicating that *CASK* regulates postnatal brain growth in a non-cell autonomous manner. Using biochemical assay, we also demonstrate that CASK can interact with metabolic proteins. *CASK* knockdown in human cell lines cause reduced cellular respiration and *CASK*^(+/-)^ mice display abnormalities in muscle and brain oxidative metabolism, suggesting a novel function of CASK in metabolism. Our data implies that some phenotypic components of *CASK* heterozygous deletion mutation associated disorders represent systemic manifestation of metabolic stress and therefore amenable to therapeutic intervention.

**Electronic supplementary material:**

The online version of this article (doi:10.1186/s40478-016-0295-6) contains supplementary material, which is available to authorized users.

## Introduction

*CASK* is an evolutionarily conserved gene which encodes for a member of the membrane-associated guanylate kinase (MAGUK) protein family [31]. In mammals CASK was discovered due to its ability to bind to the cytosolic tail of neuronal adhesion molecules neurexins and therefore is primarily identified as a scaffolding protein at the neuronal synapse [21]. However, *CASK* deletion does not alter synapse formation in C. elegans [25], *Drosophila* [56] or mouse [3]. CASK ortholog lin-2 was identified in C. elegans as early as 1980 and was found in screens for cell lineage specificity rather than synaptic function [16, 24]. Although *CASK* is essential for survival in mouse, detailed electrophysiological analysis on *CASK* null mice failed to uncover changes in core neuronal functions such as membrane excitability, calcium-dependent pre-synaptic release or post-synaptic receptor organization [3]. In fact, *CASK* evolved before the emergence of the nervous system [31, 43] and is present in tissues from all three germ layers in mammals [21, 60]. Within the brain, *CASK* is expressed by neurons and non-neuronal cells such as oligodendrocytes [2] and astrocytes [36]. The phylogenetic data and tissue distribution of CASK, together with the phenotypes of *CASK*-mutated animal models, strongly suggest that CASK may have an important but currently ill-defined non-synaptic function(s). In fact, CASK has been noted to play a role in wide variety of cellular functions including transcription regulation [27], insulin signaling and secretion [67, 72], and cancer biology [68].

Subjects with mutations in *CASK* exhibit autistic traits, intellectual disability [20], Ohtahara syndrome [51], infantile spasms [40], FG syndrome [48], mental retardation and microcephaly with pontine and cerebellar hypoplasia (MICPCH) [45, 47, 64]. In addition, *CASK* mutations are associated with growth retardation, optic nerve hypoplasia/ atrophy, epilepsy, sensorineural deafness and hypotonia often resulting in scoliosis [8, 42]. This symptomatology is strikingly similar to that of metabolic diseases [38]. Mitochondrial respiratory chain defects and mutations in mitochondrial proteins are often associated with cerebellar hypoplasia [14, 34]. Mitochondrial diseases frequently affect the optic nerve and auditory sensory circuits (reviewed in [9, 55]). Moreover, epilepsy and hypotonia are primary manifestations in mitochondrial encephalopathies [30, 61]. Syndromes associated with *CASK* mutations, e.g. Ohtahara syndrome, can also arise from respiratory chain defects [10, 69]. Recently, we found that CASK interacts with mitochondrial proteins in *Drosophila melanogaster*, pointing to a potential role of CASK in the regulation of metabolism [44].

A large component of *CASK*-associated pathology develops postnatally, which has been reported by some authors as postnatal microcephaly or even atrophy [41]. Consistent with this idea, the brain of *CASK* null mice is normal at birth and properly laminated, and no defect in synapse formation is detectable [3]; deletion of *CASK* does, however, lead to an increased apoptotic rate in the mouse brain by 3-folds [3], which might easily explain the postnatal microcephaly. Since *CASK* constitutive knockout mice die within a few hours after birth, further analysis has not been possible.

Here, we demonstrate that the cerebellar hypoplasia caused by *CASK* heterozygous deletion mutation is not due to the loss of *CASK* gene specifically in cerebellar neurons. In fact, some of the MICPCH phenotypes may be primarily due to a non-neuronal function of *CASK*. Mice that are heterozygous for *CASK* deletion in neurons appear normal, but the constitutive whole-body heterozygous knockout (*CASK*^(+/-)^) female mice phenocopy the human disease state, indicating that MICPCH occurs due to an overall haploinsufficiency of the *CASK* gene. Surprisingly, the observed microcephaly in *CASK*^(+/-)^ mice is not simply due to loss of only the *CASK* null cells i.e. it occurs in a non-cell autonomous manner. We further demonstrate that CASK interacts with mitochondrial proteins and that *CASK*^(+/-)^ mice exhibit metabolic defects. Knocking down *CASK* expression in a human cell line recapitulates these defects. These findings indicate that mammalian *CASK* has a novel role in metabolic regulation which is crucial for postnatal brain growth in mammals.

## Materials and methods

### Generation of *CASK*^(+/-)^ heterozygous and *CASK* neuronal knockout mice

*CASK*^(+/floxed)^ female mice were crossed with male mice carrying a transgenic Cre recombinase controlled by the zona pellucida 3 promoter [33]. The F1 generation offsprings were genotyped to identify female mice carrying the Cre recombinase and the floxed *CASK* gene. These female mice were crossed with wild-type C57Bl6 male mice to obtain female *CASK*^(+/-)^ mice. Crossings were continued with wild-type male mice to eliminate the Cre transgene. Colonies were maintained as *CASK*^(+/-)^ females. *CASK* neuronal knockout mice were generated by crossing the *CASK*^(+/floxed)^ female mice with a mouse line expressing Cre recombinase under the control of synapsin 1 promoter [71]. Male mice from the F1 generation bearing the Cre transgene and floxed *CASK* were analyzed. A table providing information on all mice used is shown in Additional file 1: Figure S1A.

### Generation of *CASK* knockdown cells

HEK 293 cells were transduced with PLKO lentiviral particles carrying *CASK* shRNA or empty lentiviral particles. Experiments were performed 96 h post-transduction. Cell homogenates were analyzed for *CASK* expression using quantitative immunoblotting.

### Mitochondrial respiration measurements

Mitochondrial oxygen consumption was measured in whole brain homogenates using the conventional Clark electrode assay as previously described [11]. Briefly, total respiration was measured in a buffer containing 0.3 M mannitol, 10 mM KCl, 5 mM MgCl_2_, 10 mM KH_2_PO_4_ and 1 mg/ml BSA (pH 7.4) in a water-jacketed cell magnetically stirred at 37 °C (Hansatech instruments, Norfolk, UK). Oxygen consumption rates were measured both in the presence and absence of potassium cyanide (KCN) to assess the rate of KCN sensitive respiration.Oxygen consumption measurements from HEK 293 cells were done as previously described [58, 59]. Briefly, HEK 293 cells were trypsinized and harvested in a sucrose-containing buffer (25 mM Tris–HCl, 10 mM K_2_HPO_4_ and 150 mM sucrose (pH 7.4)). Oxygen consumption in cell suspensions were measured in a water-jacketed cell magnetically stirred at 37 °C (Hansatech instruments, Norfolk, UK).

### Immunoblotting

Samples were separated by 10 % SDS-PAGE, transferred to nitrocellulose, blocked in 5 % skimmed milk for 2 h and incubated with primary antibody for 1 h followed by incubation with secondary antibody (1:5000 dilution) for 30 min at room temperature. The chemiluminescent signal was detected via the enzymatic reaction using ECL detection reagents (Amersham) and visualized on ChemiDoc (Biorad). For quantitative immunodetection, the blots were incubated with a fluorescent secondary antibody (Alexa 488) for 30 min at room temperature. The primary antibodies used included anti-ATP synthase subunit β (MitoSciences MS503, 1:1000), anti-synaptophysin (Sigma, 1:1000), anti-tubulin (DSHB, 1:1000), anti-CASK (Neuromab, 1:1000), and anti IDH (AssayBiotech, 1:1000).

### Energy expenditure and respiratory exchange ratio measurements

The energy expenditure and respiratory exchange ratios were measured over 48 h using the indirect calorimetry (TSE Systems, Chesterfield, MO). Energy expenditure data were expressed relative to free fat mass and presented as mean ± SEM. The TSE system is equipped with beams pointing in the horizontal (X,Y) and vertical (Z) directions. As mice break these beams, the LabMaster software sums this movement, which is translated into cage activity expressed as meters/hr.

### Protein quantitation

All protein quantitations were done using the Coomassie Bradford reagent from Biorad, following the manufacturer’s instruction.

### Brain sectioning and immunofluorescent staining

Animals were deeply anesthetized to avoid any pain and sacrificed mechanically either by decapitation or exsanguination. All animal procedures were performed in accordance with the Virginia Tech guidelines for use and care of laboratory animals. Three-month-old mature adult mice were deeply anaesthetized using isoflurane, and mice hearts were cannulated and perfused with PBS (exsanguination) followed by 4 % paraformaldehyde (PFA). The perfused mice were decapitated and brains were dissected out and fixed in 4 % PFA overnight. Brains were cryopreserved by incubation in 30 % sucrose solution for 48 h. For sectioning, brains were embedded in CryoTek™ and 20 μm-thick cortical sections were generated using a cryostat (IEC). The cortical sections were immunostained as floating sections. Sections were permeabilized with 0.025 % Triton X-100 followed by blocking with 5 % goat serum. Sections were stained with primary antibodies for two hours followed by secondary antibody (1:500) incubation for 30 min. Finally, sections were mounted on slides using PermaFluor™ mounting medium (Thermo Scientific) and coverslips were sealed using nail polish.

### Fatty-acid and glucose oxidation assays

Fatty-acid oxidation was assessed in the mouse brain, red and white gastrocnemius muscle and quadriceps femoris muscle by measuring and summing ^14^CO_2_ production and ^14^C-labeled acid-soluble metabolites from the oxidation of [1-^14^C]-palmitic acid (American Radiolabeled Chemicals, St. Louis MO) respectively. Briefly, samples were incubated in 0.5 μCi/ml of [1-^14^C]-palmitic acid for 3 h. Media was then removed and exposed to 45 % perchloric acid for 1 h to liberate ^14^CO_2_, which was trapped in a tube containing 1 M sodium hydroxide (NaOH). The NaOH was then placed into a scintillation vial with 5 ml scintillation fluid added. The vial was then placed on a scintillation counter (LS 4500, Beckman Coulter) and counted for the presence of ^14^C. Acid soluble metabolites were determined by collecting the acidified media and measuring ^14^C content. Glucose oxidation was assessed by measuring ^14^CO_2_ production in a similar manner to fatty acid oxidation with the exception that [U-^14^C]-glucose was substituted for [1-^14^C]-palmitic acid.

### Body composition measurement

Body composition was measured using Burker mini spec LF90, as previously described [39].

### Glutathione-*S*-transferase (GST) pull down assay

GST-fusion CASK and GST were expressed in the BL21-DE3 strain of *E. coli* and purified by affinity chromatography on a glutathione–Sepharose column (Amersham) [18]. GST-pulldowns from rat brain were performed essentially as described [37].

### Triton X-114 phase separation of homogenized mouse brain

Triton X-114 phase separation protocol was based on a previously published method [7]. Briefly, 4 % Triton X-114 (Sigma) in PBS containing protease inhibitors was mixed 1:1 with brain homogenate. The mixture was first incubated on ice for 10 min and then incubated at 37 °C for 10 min to promote separation of two phases. The sample was then centrifuged at 25 °C for 10 min at 13,000 g for separation into 1) aqueous phase on top, 2) a detergent phase in the middle, and 3) a pellet at the bottom.

### Linear glycerol gradient centrifugation

A continuous glycerol gradient (10 % - 40 %) was generated using a Gradient Master™ (BioComp Instruments, Inc.) containing 25 mM HEPES-NaOH, pH 7.2, 150 mM NaCl, 5 mM DTT, 2 mM EDTA and protease inhibitors On a 10 mL continuous glycerol gradient buffer in ultracentrifuge tubes used with SW41 Ti Rotor (Beckman Coulter), 0.5 mL of the aqueous phase from the Triton X-114 phase separation of homogenized brain was carefully layered and then centrifuged for 20 h, at 35,000 rpm at 4 °C. A total of 9 fractions (1.1 mL each) were collected using the Piston Gradient Fractionator (BioComp Instruments, Inc.). Separate tubes were used for recombinant CASK protein and protein standards in the same experiment.

### Brain subcellular fractionation and solubilization

Subcellular fractionation was performed similar to previous publication [11]. Briefly, dissected brains were rapidly transferred to ice cold homogenization buffer (i.e. 0.32 M sucrose and 20 mM HEPES pH 7.4 with protease inhibitors). The brain was homogenized in a motorized homogenizer (~20 strokes). Brain homogenates were centrifuged at 1000 g for 10 min to generate post nuclear supernatant (PNS). The PNS was centrifuged at 17,000 g for 15 min to obtain a pellet, which was subsequently washed once with homogenization buffer and then resuspended in homogenization buffer to obtain crude synaptosomes. Crude synaptosomes were subjected to ultracentrifugation (120,000 g). The supernatant was filtered through a 200 μm filter to obtain cytosolic fraction. The pellet was first solubilized in PBS containing 1 % Triton X-100 and protease inhibitors for two hours to obtain a Triton X-100-soluble fraction. The remaining pellet was further solubilized in PBS containing 1 % deoxycholic acid and protease inhibitors at 4 °C.

### Force plate actometry and rotarod treadmill experiments

For force plate analyses, the adult littermate mice per genotype performed on a force plate (Bioseb) for 5 min as described previously [22]. Ataxic index was calculated as the area traveled over the distance covered on the force plate. We also tested the ability of mice to balance on a fixed speed rotarod test (10RPM) (Ugobasile). Each mouse was given three runs each day and an average was calculated.

## Results

### Cerebellar neuron-specific deletion of *CASK* does not produce cerebellar hypoplasia

Heterozygous deletion mutations in *CASK* produce cerebellar hypoplasia [8, 45]. It has also been demonstrated that mice carrying a floxed *CASK* gene (*CASK*^floxed^) express ~ 33 % CASK compared to the wildtype littermates, and exhibit hypoplasia of cerebellar vermis [3, 45]. These data indicate that *CASK* may play a crucial role in the cerebellum. We therefore decided to use the Cre-loxP method to specifically delete *CASK* from cerebellar neurons. In order to ascertain the validity of this methodology, we first produced neuron-glia mixed cultures from P1.5 (postnatal day 1.5) *CASK*^floxed^ mice and transduced them with Cre-expressing lentivirus. Immunoblotting revealed that Cre expression specifically and completely eliminated *CASK* expression in these cultures within 96 h of virus addition. (Additional file 1: Figure S1B,C).

**Fig. 1:**
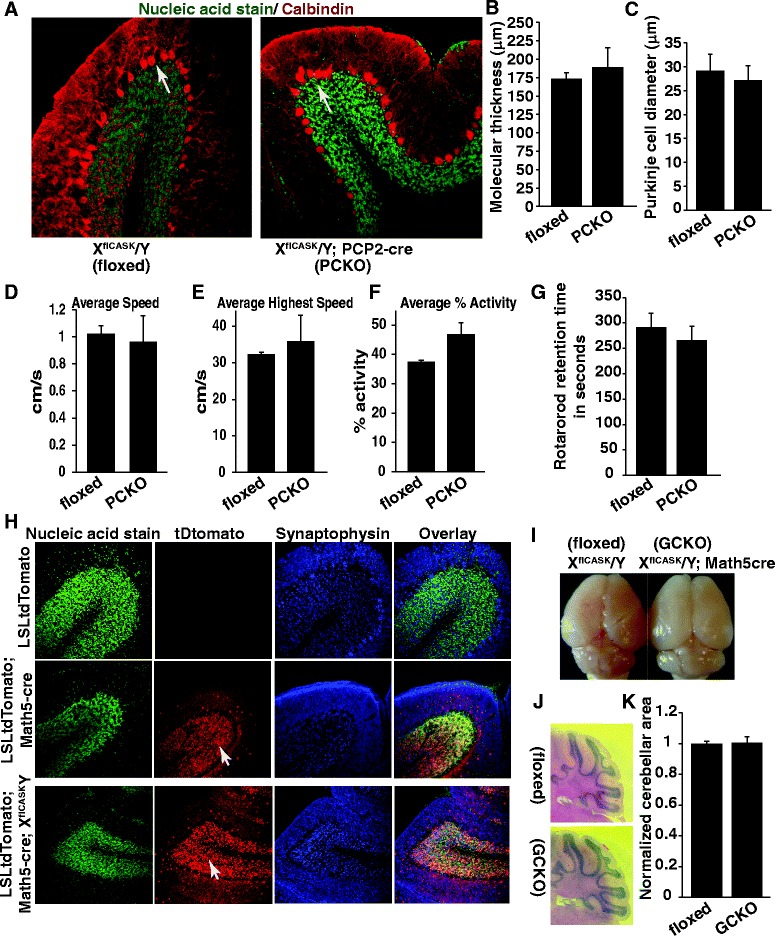
*CASK* deletion from Purkinje cells or granule cells does not alter cerebellar development. **a** Representative brain sections from two months old control mice carrying a floxed *CASK* gene (X^flCASK^/Y) and mice with *CASK* knocked out from Purkinje cells (X^flCASK^/Y; PCP2-cre); sections are stained with green (nucleic acid stain) and red (calbindin). Arrows indicate Purkinje cells. **b** Quantitation of molecular layer thickness and (**c**) Quantitation of Purkinje cell diameter; data are plotted as mean ± SEM, *n* = 3 mice. **d**, **e**, **f** Locomotion of mice analyzed in home cage using an overnight force plate experiment indicating no obvious change in locomotion. Average speed, average highest speed and average % activity are quantified and data are plotted as mean ± SEM, *n* = 3 mice. **g** Quantitation of retention time of mice on fixed speed rotarod, data are plotted as mean ± SEM, *n* = 4 mice. Floxed indicates *CASK* ^floxed^ mice and PCKO indicates Purkinje cell-specific *CASK* knockout mice. **h** Representative brain sections from two month old mice with indicated genotypes showing that *CASK* deletion from cerebellar granule cells does not lead to neuronal death or alteration in layering. LSL-tdTomato is a Cre indicator line in which a loxP-flanked stop cassette prevents expression of a red fluorescent protein; Math5-Cre mouse line has Cre recombinase expressed under the Math5 promoter. Arrows indicate inner granular layer where Cre-recombinase is expressed. Please note that the top two panels are cerebellar sections from mice with unperturbed *CASK* gene. **i** Representative brain images of two month old mice with indicated genotype. **j** Parasaggital sections of cerebellum showing that *CASK* deletion from granule cells does not lead to cerebellar hypoplasia. **k** Quantitation of cerebellar area in parasagittal sections; data are plotted as mean ± SEM, *n* = 3. Floxed indicates *CASK* ^floxed^ mice and GCKO indicates granule cell-specific *CASK* knockout mice

First, *CASK* expression was deleted from Purkinje cells -the major output neurons involved in motor coordination by crossing a *CASK*^floxed^ mouse line to a PCP2 (Purkinje cell protein 2)-Cre transgenic line [4]. Deleting *CASK* from Purkinje cells did not alter the arrangement or morphology of these cells (Fig. 1a, b, c). We analyzed the locomotion of these mice both in their home cage as well as on a rotarod. Mice lacking *CASK* in Purkinje cells exhibited no locomotory phenotype that could be interpreted as ataxic, suggesting that *CASK* is not required for the survival and functioning of Purkinje cells (Fig. 1d, e, f, g).

We next deleted *CASK* from the cerebellar granule cells by crossing the *CASK*^floxed^ mouse line to a Math5-Cre transgenic line. Math5-Cre is expressed in many different areas of brain including the hippocampus, the fourth layer of the cortex and the cerebellar cortex. In the cerebellum, cells expressing Math5-Cre give rise to the tightly packed cells in the granular layer [70]. We confirmed this distribution by crossing the Math5-Cre mouse line to an indicator mouse line that expresses tdTomato in a Cre-dependent manner [35]. Our experimental results show that Math5-Cre is expressed in granular cells as well as in a few cells in the molecular layer (Fig. 1h, middle panels). Granular cells are formed in the external granular layer during cerebellar genesis and subsequently migrate inwards. The *CASK* null granular cells are capable of this migration as indicated by their proper localization in the adult mouse cerebellum (Fig. 1h, lower row). Furthermore, we found that deleting *CASK* from the cerebellar granule cells did not alter the size of the cerebellum or layering of neurons compared to the *CASK*^floxed^ mice (Fig. 1i, j, k). No specific motor deficits were observed in any of these mice (data not shown). Our data therefore imply that although *CASK* may play an important role in cell survival in the postnatal brain, deleting it from specific neuronal subtypes is compatible with their survival. Furthermore, our data indicates that the cerebellar hypoplasia phenotype observed in *CASK* mutation subjects may not be directly related to the loss of *CASK* function in cerebellar neurons.

### MICPCH pathology is not purely neuronal in origin

**Fig. 2:**
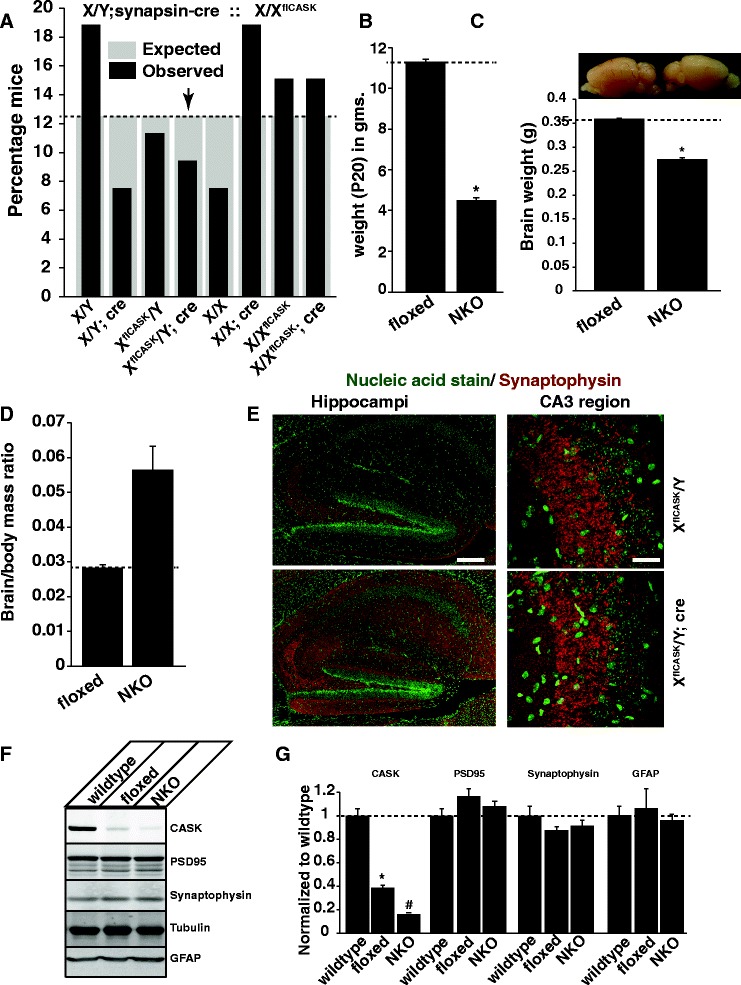
Survival and normal brain formation in *CASK* neuronal knockout mice. **a** Shows the expected (*grey bar*) and observed (*black bar*) percentage of mice with each genotype obtained from crossing the X/Y;synapsin-Cre males with X^flCASK^/X females; *n* = 62. Arrow indicates the neuronal *CASK* knockout group. **b** Weight of *CASK* ^floxed^ (floxed) and neuronal *CASK* knockout male mice (NKO) at 20 days of age. (* indicates *p* < 0.05; *n* = 5) (**c**) Representative brain halves and brain weight at 21 days of age from *CASK* (X^flCASK^/Y) (floxed) and neuronal *CASK* knockout (NKO) mice. (* indicates *p* < 0.05; *n* = 4). **d** Ratio of brain to body weight of *CASK* (X^flCASK^/Y) (floxed) and neuronal *CASK* knockout mice (NKO). (* indicates *p* < 0.05; *n* = 4). **e** Image showing hippocampus from 21 day old *CASK* ^floxed^ and *CASK* neuronal knockout mice. Green staining indicates nucleic acid and red staining indicates synaptophysin. Note that there is no significant change in the lamination and CA3 region synaptophysin staining in *CASK* neuronal knockout mice. **f** Representative Western blot showing endogenous levels of CASK, PSD95, synaptophysin and tubulin from 21 day old mice. **g** Western blot protein quantitation where data is normalized to tubulin and expressed relative to wild-type levels. Bar graphs are plotted as mean ± SEM. (* indicates *p* < 0.05; *n* = 3). Wild-type represents littermate mice with unperturbed *CASK* gene, floxed indicates *CASK* ^floxed^ mice and NKO indicates neuron-specific *CASK* knockout mice

*CASK* knockout mice exhibit perinatal lethality and heightened apoptotic rates in the brain, indicating that *CASK* is essential for *ex utero* survival of brain cells [3]. However, our cerebellum-specific deletion experiments suggest that *CASK* may not be important for cell-autonomous survival. A large body of literature suggests that CASK may have a potential synaptic function (Reviewed in [26]), abnormality in which may lead to death of brain cells. Previously, attempt to generate brain specific CASK knockout mice was done using a transgenic mice expressing cre recombinase driven by nestin promoter to delete CASK from embryonic brain. The resultant mice exhibited perinatal lethality like the constitutive *CASK* knockout mice (personal communication with Prof. Susanne Schoch McGovern). However besides neurons, nestin is also expressed in non-neuronal brain cells and even in extra-neuronal tissue which made it difficult to interpret this data [65, 66]. We have therefore generated the pan-neuronal-specific *CASK* knockout mice by crossing *CASK*^floxed^ mice [3] with a synapsin1-Cre line which expresses Cre exclusively in post-mitotic neurons [54, 71]. In parallel, we also crossed the synapsin-Cre mice with the reporter mouse line that expresses tdTomato in a Cre-dependent manner. Our experiments confirmed the previous findings that Cre-mediated recombination in these mice occurs in nearly all neurons at an embryonic stage (as early as E12.5) ([71] and Additional file 1: Figure S2A,B). Since most *CASK*-associated patients are females harboring a heterozygous mutation in *CASK*, we initially examined female mice heterozygous for deletion of *CASK* pan-neuronally. Surprisingly, female mice heterozygous for neuron-specific *CASK* deletion exhibit no major phenotype and have normal-sized brains, including the cerebellum (Additional file 1: Figure S2C,D,E,F). This observation strongly suggests that *CASK*-associated MICPCH in humans is not specifically a neuronal pathology. Consistent with this assumption, complete deletion of *CASK* from all neurons did not produce the neonatal lethality observed in the constitutive *CASK* knockout mice [3] (Fig. 2a). The *CASK* neuronal-null mice are indistinguishable from their wild-type littermates until ~ 10 - 12 days after birth and maintain mobility until 21 days after birth, indicating that they have functional synapses (Additional file 2: Movie S1). After 10 days of birth, the *CASK* neuronal-null mice begin to exhibit a slower growth rate and by ~ 20 days of age, *CASK* neuronal-null mice are less than half the size of the control *CASK*^floxed^ mice or wild-type littermates (Fig. 2b). Brain weight is also significantly reduced in the *CASK* neuronal knockout pups, although the cerebellum appears normal compared to *CASK*^floxed^ mice and wildtype mice (Fig. 2c and Additional file 1: Figure S3). True microcephaly has been defined by some studies as an adult brain which is not only small but small for body weight [13, 23]. We therefore next measured the ratio of brain weight to body weight in *CASK* neuronal-null mice. The brain weight to body weight ratio is however significantly increased in the *CASK* neuronal-knockout mice compared to the control *CASK*^floxed^ mice or wildtype mice (Fig. 2d and data not shown), suggesting that the reduction in brain weight is secondary to overall growth retardation. Brain lamination is unaltered, and the neuronal arrangement in *CASK* neuronal knockout hippocampi and cerebella are comparable to control mice (Fig. 2e and Additional file 1: Figure S3). Neuronal *CASK* knockout mice express ~ 15 % of wild-type *CASK* in the brain (Fig. 2f, g), which may be due to *CASK* expression in other cell types including astrocytes [36] and oligodendrocytes [2]. Although *CASK* is deleted from most neurons at the embryonic stage, the levels of pre-synaptic and post-synaptic markers are unchanged, consistent with the previous study that demonstrated synapse formation is unaltered in *CASK* knockout mice [3]. The level of glial fibrillary acidic protein (GFAP) is also unaltered, indicating that although *CASK* is specifically deleted in neurons, there is no reactive gliosis and the ratio of neurons to astroglia may be unaltered (Fig. 2f, g). Beginning around 17-18 days of age, these mice suffer from progressively increasing bouts of epileptic seizures and spasms, which often prove fatal before 23 - 24 days of age (Additional file 3: Movie S2). Incidentally, epileptic spasms and epileptic disorders have been demonstrated in patients with *CASK* mutations [46, 51]. It has to be however considered that the *CASK* neuronal-null mice are produced on the *CASK*^floxed^ mice background, so glial cells are generating only ~ 33 % of CASK. The strong phenotype in the *CASK* neuronal-null mice therefore may indicate a combination of effect from CASK-null neurons and CASK-hypomorphic glial cells. Altogether, these data suggest that although loss of *CASK* function in neurons contributes to some components of the phenotypic spectrum of *CASK*-associated pathology (e.g. growth retardation and epilepsy), it does not contribute to microcephaly or cerebellar hypoplasia. Many *CASK* missense mutations in human males are associated with intellectual disabilities and growth retardation in the absence of microcephaly, clearly indicating that these phenotypes are not interdependent and may represent loss of different molecular functions of *CASK* [20].

### *CASK*^(+/-)^ mice mimic the human MICPCH phenotype

**Fig. 3:**
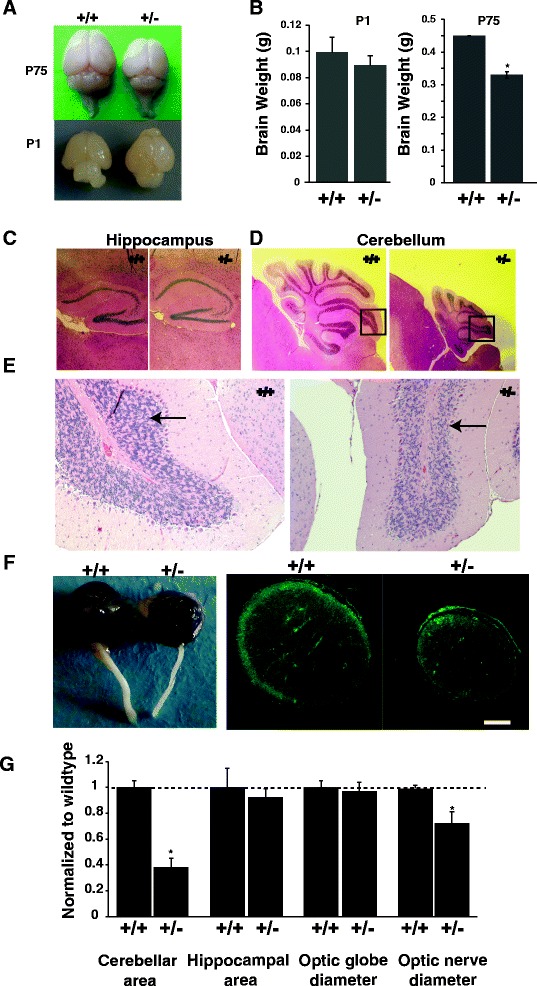
*CASK* ^(+/-)^ heterozygous mutant mice display postnatal microcephaly, cerebellar hypoplasia and optic nerve hypoplasia. **a** Representative brain images of the sex-matched *CASK* ^(+/+)^ and *CASK* ^(+/-)^ mutant littermates at postnatal day 1 and day 75 (P1 and P75), respectively. **b** Quantitation of brain weights from *CASK* ^(+/+)^ and *CASK* ^(+/-)^ mice at P1 and P75 (* indicates *p* < 0.05; *n* = 4). **c** Hematoxylin and eosin (H&E) stained sections of hippocampus derived from *CASK* ^(+/+)^ and *CASK* ^(+/-)^ mice at P75; note the hippocampi sizes are comparable. **d** H&E stained sections of the cerebellum at P75 showing pronounced cerebellar hypoplasia in *CASK* ^(+/-)^ mice relative to the *CASK* ^(+/+)^ control. **e** High magnification of the indicated square regions from panel D showing relatively fewer cells in the cerebellar folia of *CASK* ^(+/-)^ mice compared to the *CASK* ^(+/+)^ control. **f** Left panel shows representative images of the optic globe and optic nerve derived from three month old *CASK* ^(+/+)^ and *CASK* ^(+/-)^ mice . Right panel shows the optic nerve cross section stained with anti-tubulin antibody from *CASK* ^(+/+)^ and *CASK* ^(+/-)^ mice; note the decrease in optic nerve diameter . Scale bar = 100 μm. **g** Quantification of the cerebellar and hippocampal areas and the optic nerve and optic globe diameters obtained from *CASK* ^(+/+)^ and *CASK* ^(+/-)^ mice. Measurements were made using Image J software and normalized to the sex-matched wild-type littermate controls. Bar graphs are plotted as mean ± SEM; (* indicates *p* **<** 0.05; *n* = 4)

**Fig. 4:**
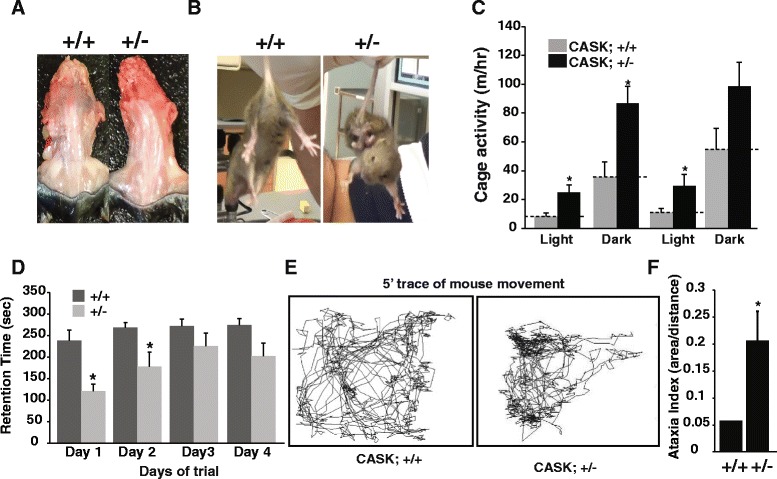
*CASK* ^(+/-)^ mice display musculoskeletal and locomotion defects. **a** Dissected torso from three month old *CASK* ^(+/+)^ and *CASK* ^(+/-)^ mice; *CASK* ^(+/-)^ mice display scoliosis. **b** *CASK* ^(+/-)^ mice display hind-limb clasping phenotype. **c** Activity of *CASK* ^(+/+)^ and *CASK* ^(+/-)^ mice measured in metabolic cages. Data plotted as mean ± SEM; (* indicates *p* < 0.05; *n* = 5) (**d**) *CASK* ^(+/-)^ mice were trained on a fixed speed rotorod and retention time noted. On each day, fresh sets of sex-and-age matched *CASK* ^(+/+)^ littermate mice were used as controls. Data are plotted as mean ± SEM; (* indicates *p* < 0.05; *n* = 5). **e** Representative 5-min trace of positions of *CASK* ^(+/+)^ and *CASK* ^(+/-)^ mice in home cage detected by a force plate. **f** Quantitation of locomotion observed on a force plate (area covered in 5 min/net distance traveled). Data are plotted as mean ± SEM; (* indicates *p* < 0.05; *n* = 5). All locomotion experiments were performed on mice between 25-30 days of age

Since *CASK*-related neurological disorders occur due to deletion of a single *CASK* allele in females, we engineered a *CASK*^(+/-)^ mouse line to model the neurodevelopmental defects. (Methods section and Additional file 1: Figure S4). Similar to the *CASK*^(-/-)^ complete knockout mice, the brains of *CASK*^(+/-)^ knockout mice were indistinguishable from their wild-type littermates at birth (Fig. 3a) [3]. However, pronounced microcephaly was observed beginning one week after birth, and the brains of adult *CASK*^(+/-)^ females were ~ 25 % smaller in weight compared to sex-matched wild-type littermate controls (Fig. 3a, b). The hematoxylin and eosin (H&E) staining of brain sections showed no significant change in hippocampal size and layering of cells in the *CASK*^(+/-)^ mice (Fig. 3c, g), but a marked decrease in cerebellar size (Fig. 3d, g) and number of cells in the cerebellum (Fig. 3e) was observed in the *CASK*^(+/-)^ mice compared to the *CASK*^(+/+)^ mice, suggesting disproportionate cerebellar hypoplasia. In contrast to lissencephaly [17], the *CASK*^(+/-)^ cerebellum exhibited normal layering of cells (Fig. 3c, d, e). Examination of the cerebella of 5-day-old *CASK*^(+/-)^ mice revealed that the thickness of the external granular layer (where granule cells are formed) remains unchanged (Additional file 1: Figure S5A,B). These data are consistent with our previous findings that lack of CASK increases cell loss by apoptosis rather than by producing any deficiency in the formation of cells [3]. Similar to *CASK* heterozygous deletion mutation patients, the *CASK*^(+/-)^ mice displayed optic nerve hypoplasia (ONH); the optic nerve diameter was significantly reduced compared to sex-matched *CASK*^(+/+)^ littermate controls (Fig. 3f, g). Although we observed a trend towards microphthalmia in *CASK*^(+/-)^ mice, the optic globe size difference did not reach a statistical significance (Fig. 3g). ONH is frequently accompanied by malformation of the brain midline [5], but we did not observe such an anomaly in the CASK^(+/-)^ mice (Additional file 1: Figure S6A), suggesting that ONH associated with *CASK* heterozygous deletion mutation has a distinct pathology. Similar to *CASK* patients, *CASK*^(+/-)^ mice display scoliosis with a high degree of penetrance (~85 %) (Fig. 4a). *CASK*^(+/-)^ mice also readily display a hind-limb clasping phenotype (Fig. 4b) that is indicative of neurological and motor dysfunction [19]. Atypical limb clasping phenotype involving four-limb clasping was also frequently observed. We did not find any anatomical aberrations in peripheral nerves such as myelination defects (data not shown) or neuromuscular junction abnormalities (Additional file 1: Figure S6B), which indicates that both hypotonia and the hind-limb clasping phenotype most likely originate from central nervous system impairment. *CASK*^(+/-)^ mice also display increased diurnal activity relative to their sex-matched wild-type littermate controls (Fig. 4c) and are ataxic. *CASK*^(+/-)^ mice fall off a rotarod much earlier (<2 min) than control mice (~6 min) due to ataxia in the first two days of training. Upon repeated training, however, these mice exhibit motor learning (Fig. 4d). Ataxia in mice can also be visualized by tracking movements within their home cage using a force plate. We observed that the *CASK*^(+/-)^ mice traveled a longer distance to cover the same area compared to the *CASK*^(+/+)^ mice (Fig. 4e, f), a parameter that is indicative of a higher ataxic index [22]. In sum, these data suggest that *CASK*^(+/-)^ mice recapitulate multiple phenotypes observed in patients with MICPCH and therefore MICPCH represents heterozygous loss of *CASK* function (i.e. functional haploinsufficiency).

### *CASK* deletion affects cell number in a non-cell-autonomous manner

**Fig. 5:**
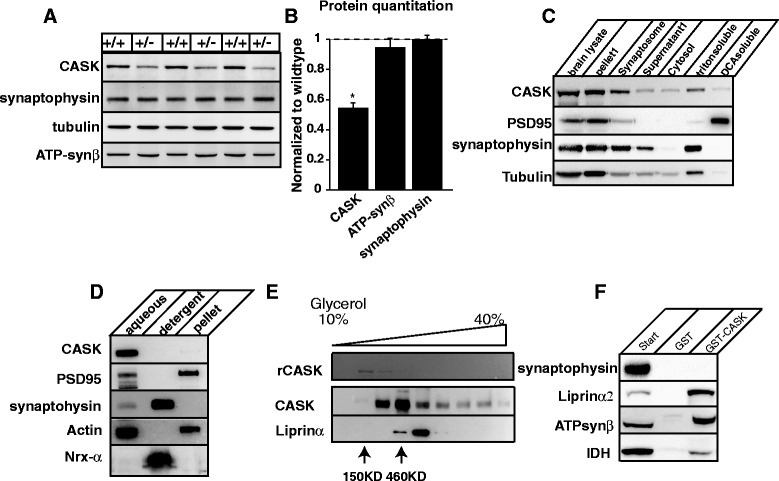
CASK is present in cytosol as a part of large protein complexes. **a** Western blot showing endogenous level of CASK, synaptophysin, ATP synthase β subunit and tubulin from *CASK* ^(+/+)^ and *CASK* ^(+/-)^ mutant mice (*n* = 3). **b** Western blot quantitation in *CASK* ^(+/-)^ mice relative to *CASK* ^(+/+)^ mice. Data is normalized to tubulin and expressed relative to wild-type levels. Bar graphs are plotted as mean ± SEM. (* indicates *p* < 0.05; *n* = 3). **c** Wild-type mice brains were initially fractionated to separate synaptosomal and cytosolic fractions. The synaptosomal fraction was solubilized initially in Triton X-100 and then in deoxycholic acid (DCA). Samples were blotted for the indicated antigen. **d** Wild-type mouse brain homogenate was solubilized in Triton X-114 at 4 °C, samples were warmed up to 37 °C and centrifuged to separate into an aqueous phase, a detergent phase and a pellet. Equal dilutions of each phase were blotted for the indicated proteins. **e** Aqueous phase (after Triton X-114 phase separation) from brain containing CASK was carefully layered on a 10-40 % continuous glycerol gradient. Following ultracentrifugation, samples were blotted for CASK and liprin-α3. Fig. also shows pure recombinant CASK. Arrows indicate the fractions where protein standards of 150 kDa and 460 kDa were observed. **f** GST-pulldown assay performed from rat brain homogenates using either full-length GST-CASK or GST (negative control) immobilized on glutathione resin. Synaptophysin (negative control); liprin α2 (positive control); ATPsynβ (ATP synthase β subunit); and IDH (isocitrate dehydrogenase)

Although *CASK*^(-/-)^ homozygous deletion has been shown to enhance the rate of apoptosis by nearly 3-fold in the brain [3], deleting *CASK* specifically from neuronal cells revealed that *CASK* is not essential for neuronal survival in a cell-autonomous manner (Figs. 1 & 2). We therefore next sought to determine if the microcephaly in *CASK*^(+/-)^ mice was due to a reduction in cell number in a non-cell-autonomous manner. *CASK* is an X-linked gene and hence subject to random inactivation, therefore brains from female mice with a *CASK*^(+/-)^ genotype are mosaic for CASK expression, with ~ 50 % of brain cells not expressing *CASK*. Cell death specifically of *CASK*-deleted brain cells in a *CASK*^(+/-)^ mouse brain would result in the majority of surviving cells being CASK-positive (Additional file 1: Figure S7A,B,C). We tested this hypothesis by performing a quantitative immunoblotting experiment from *CASK*^(+/+)^ and *CASK*^(+/-)^ mouse brain homogenates. Total CASK levels were reduced by ~ 50 % in the brain of *CASK*^(+/-)^ mice compared to the *CASK*^(+/+)^ mice (Fig. 5a, b), but no significant change was observed in the level of a synaptic marker (synaptophysin) or a generic cell marker (ATP synthase β subunit), demonstrating that *CASK* null cells are equally viable in the brain of *CASK*^(+/-)^ mice. These data suggest that the observed microcephaly in *CASK*^(+/-)^ mice is not due to the cell-autonomous loss of *CASK* null cells.

### CASK is an intracellular cytosolic protein and interacts with mitochondrial proteins

CASK is thought to be either completely or mainly membrane-anchored protein [21, 28]. Deletion of a signaling molecule at the membrane may explain a non-cell autonomous effect. We therefore decided to re-examine the cellular localization of CASK to confirm its presence at the membrane. We fractionated the wild-type mouse brain and looked for the solubility of CASK compared to PSD95, another MAGUK protein which is known to be membrane anchored [28, 63]. As expected, we found that PSD95 is completely membrane-anchored and is solubilized only with deoxycholic acid treatment, whereas a significant amount of CASK is also present in the cytosolic fraction isolated from the mouse brain, indicating that CASK is a soluble cytosolic protein (Fig. 5c). In this regard our finding is consistent with a previous report [28]. To further explore CASK’s cellular localization, we performed a Triton X-114 phase separation experiment. Triton X-114 solution is homogenous at 0 °C but separates into a detergent and aqueous phase at 20 °C, allowing for separation of hydrophilic cytosolic proteins from amphiphilic transmembrane proteins [6]. Since Triton X-114 is a mild non-ionic detergent, it is unlikely to disrupt strong interactions [6]. Interestingly, most of the CASK protein partitioned in the aqueous phase indicating that CASK is not tightly membrane-anchored and predominantly a cytosolic protein (Fig. 5d). In contrast to CASK, only a minor fraction of PSD95 is present in the aqueous phase (Fig. 5d). These results suggest that a large fraction of CASK protein may be cytosolic in the mouse brain, and in this aspect CASK differs substantially from other MAGUK proteins such as PSD95, which is tethered to the membrane to orchestrate cell-to-cell signaling.

In *Drosophila,* it is known that CASK interacts with different protein complexes in different cell types [44]. We therefore sought to determine if in the mammalian brain, CASK is also part of large protein complexes. We first expressed and purified full-length recombinant rat CASK (rCASK). CASK is a monomeric protein based on the gel exclusion chromatography experiments (data not shown). After glycerol gradient centrifugation, the rCASK (MW 120 kDa) protein was present in the second fraction along with a 150 kDa standard marker, as expected. Since rCASK migrates at ~ 120 kDa on SDS-PAGE, this confirms that rCASK is a monomer. In contrast, a large portion of the mouse brain cytosolic CASK sedimented in the fourth fraction along with a 460 kDa marker, indicating that when in cytosol, CASK is likely a part of a multi-protein complex (Fig. 5e). Surprisingly, small amounts of CASK were found to sediment in all fractions unlike the other well-known scaffolding protein e.g. liprin-α3 which displayed a well-defined sedimentation fraction indicating that the mouse brain CASK may be present in many complexes with molecular weights larger than 500 kDa (Fig. 5e).

Since CASK interacts with mitochondrial proteins in *Drosophila* [44], we next sought to determine if CASK also interacts with mitochondrial proteins in mammals. Using immobilized full-length recombinant CASK protein covalently attached to AminoLink™ agarose resin, we performed an affinity chromatography experiment using rat brain lysate and identified several CASK-interacting mitochondrial proteins from rat brain (unpublished data). To test the specificity of these interactions, we performed a pull-down assay with a GST (glutathione-S-transferase)-tagged CASK and found that CASK interacts with the mitochondrial ATP synthase β subunit and isocitrate dehydrogenase (IDH) (Fig. 5f).

### *CASK*^(+/-)^ heterozygous mice show aberrant metabolism

**Fig. 6:**
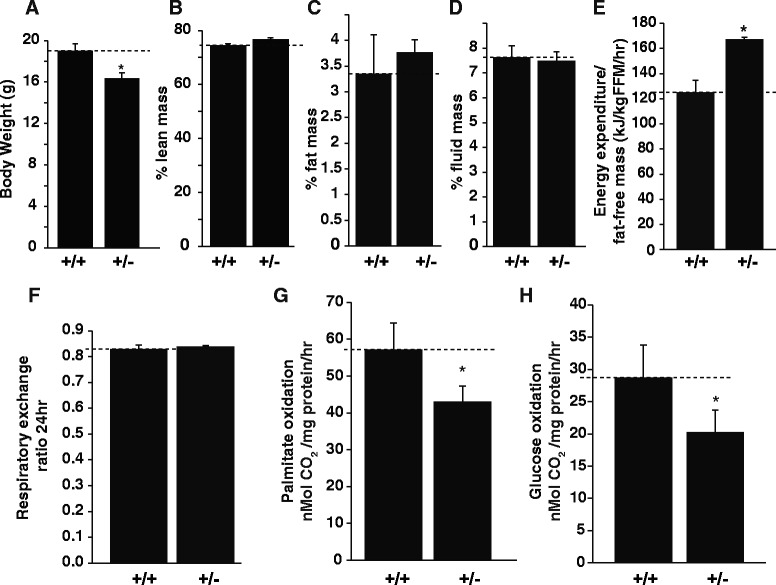
*CASK* ^(+/-)^ mutant mice exhibit growth retardation and skeletal muscle metabolic defects. Comparison of (**a**) body weight, (**b**) percentage lean mass, (**c**) percentage fat mass and (**d**) percentage fluid mass. All comparisons are presented as mean ± SEM, (* indicates *p* < 0.05; *n* = 5). **e** Energy expenditure/fat-free mass/hr in a 24-h period from *CASK* ^(+/-)^ mice compared to sex-matched *CASK* ^(+/+)^ littermate control. Data is presented as mean ± SEM (* indicates *p* < 0.05; *n* = 5). **f** Respiratory exchange ratio from *CASK* ^(+/-)^ mice compared to sex-matched *CASK* ^(+/+)^ littermate control. Data is presented as mean ± SEM. **g** Fatty acid oxidation rate and (**h**) glucose oxidation rate measured in skeletal muscle homogenates from *CASK* ^(+/+)^ and *CASK* ^(+/-)^ sex-matched littermate mice. Bar graphs are plotted as mean ± SEM. (* indicates *p* < 0.05; *n* = 5). All experiments were performed on one month old mice

Since mitochondrial proteins co-precipitates with CASK in *Drosophila* [44] and mice, we hypothesized that CASK may play a role in regulating metabolism. To test this hypothesis, we characterized the consequences of *CASK* deletion on mitochondrial function and whole body metabolism. Our results showed that the one-month old *CASK*^(+/-)^ mice weigh less (a small but statistically significant difference) than the sex-matched littermate controls (Fig. 6a), however there is no significant change in their lean body mass, fat weight or fluid weight, indicating an overall growth retardation (Fig. 6b, c, d). Surprisingly, we also found that *CASK*^(+/-)^ mice expend significantly more energy at rest (Fig. 6e), which is likely attributable to ataxia and imbalance in these mice. It is known that an increase in muscle movement increases the respiratory exchange ratio (CO_2_ production/O_2_ uptake) [49], so we next examined the respiratory exchange ratio (RER). The RER of *CASK*^(+/-)^ mice was indistinguishable from the *CASK*^(+/+)^ littermate controls, despite an increased energy expenditure (Fig. 6 f).

Because some studies indicate that CASK plays a role in endocrine function such as insulin release [72], it is important to consider the fact that an overall measure of organism or organ metabolism may not be indicative of CASK’s direct involvement in cellular metabolism. We therefore examined the rate of metabolic fuel oxidation in skeletal muscle homogenates isolated from *CASK*^(+/-)^ mice compared to the sex-matched *CASK*^(+/+)^ littermate controls. Interestingly, we observed that the skeletal muscles from *CASK*^(+/-)^ mice oxidize significantly lower amounts of fatty acids (Fig. 6g) and glucose (Fig. 6h) compared to the *CASK*^(+/+)^ mice, suggesting that CASK is involved in the regulation of cellular substrate metabolism.

### CASK regulates mitochondrial respiration and oxidative metabolism

**Fig. 7:**
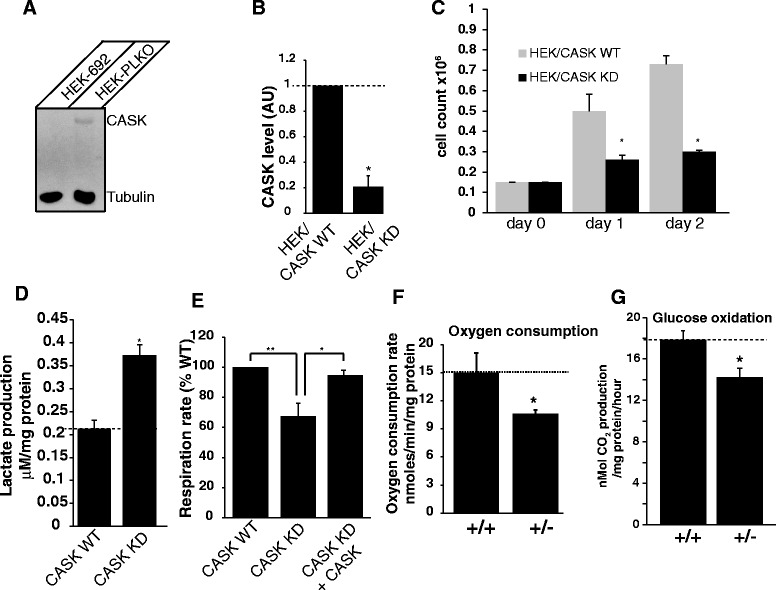
Reduction of CASK expression in cells and brain reduces oxidative metabolism. **a** Representative Western blot showing endogenous *CASK* expression in human embryonic kidney (HEK293-PLKO) cells as well as *CASK* knockdown in HEK293 cells using shRNA692 (HEK-692). **b** Quantitation of *CASK* knockdown (KD) in HEK293 cells using shRNA692 (* indicates *p* < 0.05; *n* = 3) (WT = wildtype). **c** Hemocytometer cell count in *CASK* knockdown (KD) HEK293 cells compared to wild-type control cells (WT). Bar graphs are plotted as mean ± SEM (*n* = 4; * indicates *p* < 0.05). **d** Lactate measurement from the culture media of wild-type control (WT) or *CASK* knockdown (KD) HEK293 cells. Bar graphs are plotted as mean ± SEM (* indicates *p* < 0.05; *n* = 3). **e** Total cellular respiration rate in *CASK* knockdown (KD) HEK293 cells and *CASK* knockdown HEK293 cells overexpressing recombinant rat CASK protein (KD + CASK) compared to the wild-type control cells (WT). Respiration is normalized to total protein levels (*n* = 4; * indicates *p* < 0.05, ** indicates *p* < 0.01). **f** Oxygen consumption rate measured in brain homogenates isolated from one month old *CASK* ^(+/+)^ and *CASK* ^(+/-)^ mice. Respiration rate is normalized to the total protein content (* indicates *p* < 0.05; *n* = 4). **g** Glucose oxidation rate measured in brain homogenates isolated from *CASK* ^(+/+)^ and *CASK* ^(+/-)^ sex-matched littermate mice (* indicates *p* < 0.05; *n* = 4)

The human embryonic kidney 293 (HEK293) cells express *CASK* endogenously (Fig. 7a). To determine if *CASK* regulates mitochondrial function, we performed *CASK* knockdown experiments in HEK293 cells using two different shRNAs (out of 5 shRNAs tested; Additional file 1: Figure S8) that showed > 50 % reduction in *CASK* expression. Specifically, the shRNA 692 and shRNA 690 showed ~ 85 % and ~ 60 % *CASK* knockdown respectively in HEK293 cells compared to the vector transfected control cells (Fig. 7a, b and Additional file 1: Figure S9A,B). Knocking down *CASK* expression using shRNA 692 significantly reduced the number of HEK293 cells and increased lactate production (Fig. 7c, d). Total cell respiration was measured using a conventional Clark oxygen electrode [58], and was found to be significantly reduced in *CASK* knockdown HEK293 cells compared to the control cells (Fig. 7e). Moreover, expressing rat CASK in *CASK* knockdown HEK293 cells rescued the defect in respiration demonstrating the specificity of the phenotype (Fig. 7e). The decrease in respiration and cell numbers was also replicated using the shRNA 690 that showed > 50 % *CASK* knockdown, but not with other shRNAs (i.e shRNAs 691, 693 and 694) that showed < 50 % *CASK* knockdown indicating a dose dependency of this phenotype (Additional file 1: Figure S9). Taken together, these results suggest that mammalian CASK is a regulator of mitochondrial respiration.

Since the neonatal brain is highly susceptible to defects in metabolism, we next examined the effects of *CASK* deletion on oxidative metabolism in the brain. We adopted the same strategy as in muscles by using the brain homogenates to rule out any indirect effect/s of *CASK* deletion. Whole brain homogenates have been shown to exhibit a more tightly coupled respiration than isolated mitochondria [62]; and we also recently found that respiration can be measured more effectively from whole brain homogenates [11]. The rate of endogenous respiration in whole brain homogenates from *CASK*^(+/-)^ mice was significantly reduced (~25 % decrease) compared to the *CASK*^(+/+)^ brain homogenates (Fig. 7f), further suggesting that CASK is a regulator of mitochondrial respiration in the brain. Since glucose is the major source of fuel in the brain, we next asked whether glucose oxidation was altered in the brain of *CASK*^(+/-)^ mice. We found a significant reduction (~20 % decrease) in the rate of glucose oxidation in *CASK*^(+/-)^ mice brain compared to the *CASK*^(+/+)^ brain (Fig. 7g), suggesting that CASK regulates oxidative metabolism in the brain.

## Discussion

XLID affects males at a significantly higher rate than females because males have a single X-chromosome [50]. In females, X-linked mutations create a mosaic brain due to random X-inactivation, with ~ 50 % of brain cells expressing the mutant gene. This results in a relatively milder phenotype, as exemplified by the *doublecortin* mutations [1]. Most *CASK* deletion mutation patients are females, indicating that *CASK* expression is essential in the majority of cells for adequate brain function.

*CASK* heterozygous deletion mutation patient phenotypes have been classified as MICPCH or pontocerebellar hypoplasia. Here, we demonstrated that cerebellar hypoplasia associated with *CASK* mutations may not be a local neuronal pathology. One possible explanation for the disproportionate cerebellar hypoplasia could be the time of onset of this disorder; *CASK* heterozygous deletion mutations lead to aberrant postnatal brain growth and since cerebellar growth is largely postnatal, it may be affected disproportionately. In fact, it is known that although nutritional deprivation during postnatal brain development inhibits overall brain growth, it affects cerebellum disproportionately [57].

Consistent with the notion that CASK is not involved in core neuronal functions [3], we demonstrate that neuronal-specific *CASK* deletion does not alter neuronal migration or survival. Although the neuron-specific *CASK* heterozygous knockout mice did not exhibit any major phenotype, the complete neuronal *CASK* knockout mice exhibited pronounced growth retardation and epilepsy, and both are symptoms of *CASK*-related syndromes. Since, the *CASK* neuronal-null mutants are made on a hypomorphic background, at present it is not possible to specifically point out the consequences of deleting *CASK* only from neurons. However, our data suggests that the disruption of neuronal *CASK* function does contribute to the overall *CASK* mutation-related phenotypes.

Phenotypes associated with *CASK* heterozygous deletion mutation in humans may represent a cumulative loss of *CASK* function in different brain cell types. Since CASK interacts with multiple synaptic proteins, it is presumed to possess a synaptic function [52]. Our recent proteomic screening experiments in *Drosophila melanogaster* confirms the synaptic protein interactions of CASK, but also revealed a hitherto unknown interaction of CASK with mitochondrial proteins [44]. In agreement with our previous findings, we demonstrate here that mammalian CASK also interacts with certain metabolic/mitochondrial proteins. Since a significant amount of CASK was found to be cytosolic in the mouse brain, we speculate that these interactions may be transient and taking place in the cytosol. Interestingly, the cytosolic interaction and phosphorylation of nuclear encoded mitochondrial proteins with other kinases have been shown to affect their import into mitochondria [53]. Reducing *CASK* expression in a cellular or animal model alters mitochondrial function, including oxygen consumption and fuel metabolism. Since the cell biological role of neuroglial cells and neurons are considerably different, specific deletion of *CASK* in each cell type is likely to produce different phenotypes. A metabolic function of CASK could also explain the phenotypic differences observed in multiple animal models of CASK deficiency, including the vulval phenotype in worms and a defective locomotion phenotype in flies, both of which are known to be associated with abnormal metabolism [12, 29]. In mammals, the high metabolic demand of the growing neonatal brain could explain the lethality and postnatal onset of microcephaly and cerebellar hypoplasia in cases of CASK deficiency. It is possible that the species-specific phenotypic differences represent a difference in the metabolic requirements of each specific nervous system rather than an evolutionary difference in the molecular function of CASK.

How does a cell-autonomous metabolic function of CASK translate into the reduction of cell numbers in a non-cell-autonomous manner? Although it may be assumed that a significant reduction in the mitochondrial function would be deleterious to the survival of neurons, the extent to which the energetic function is compromised may further determine whether neurons may survive abnormally or undergo cell death. In human mitochondrial encephalopathies, the symptoms typically do not manifest until two years of age, indicating adequate survival of neurons during development [15]. Importantly, the central nervous system pathology like stroke episodes in mitochondrial encephalopathies may also be related to an aberrant extracellular signaling e.g. nitric oxide mediated signaling [15]. The non-cell autonomous systemic manifestations of mitochondrial stress are gradually getting recognized [32]. It is possible that CASK deficiency modulates one or more metabolic pathway/s which are critical for the secretion of a factor(s) essential for cellular survival. Conversely, it is also possible that CASK deficiency leads to the release of toxic intermediates, resulting in the loss of cells in a non-cell autonomous manner. Mitochondrial function in the brain is not only critical to ATP production but also in regulating amino-acid metabolism, which governs neurotransmitter production. An altered milieu of different neurotransmitters in the brain may have profound implications on neurodevelopment. It can therefore be hypothesized that the postnatal microcephaly associated with *CASK* heterozygous deletion mutation may be caused by altered levels of one or more extracellular factors. Although much remains to be done to identify the specific players involved, the most exciting aspect of this study is the possibility that a metabolic intervention may potentially prevent some of the postnatal consequences associated with *CASK* deletion mutation.

## Conclusions

Taken together our results suggest that lack of neuronal CASK function contributes to the phenotypic spectrum of *CASK* linked pathologies, however MICPCH is not purely neuronal in origin. Therefore, constitutive heterozygous *CASK* knockout females are better animal model to investigate MICPCH. Our data further indicates that although CASK is a cytosolic protein and may perform cell-autonomous function in regulating metabolism, *CASK*-linked microcephaly occurs in a non-cell autonomous manner which involves loss of both CASK positive and CASK null cells. Finally, the observed cerebellar hypoplasia is not a local neuronal pathology; disproportionate cerebellar hypoplasia may simply represent timing of the disorder which has a large postnatal component. It is known that nutritional deprivation during the postnatal brain growth spurt specifically affects the cerebellum. Therefore, our findings also provide a valid framework for the investigation of non-CASK related cerebellar hypoplasia.

### Ethical statement

All procedures performed in studies involving animals were in accordance with the ethical standards of the VirginiaTech institutional animal care and use committee.

## Additional files

Additional file 1: Figure S1. Description of mouse lines used and effect of cre expression on floxed CASK gene. **Figure S2.** CASK deletion from neurons and characterization of female heterozygous mice with neuronspecific CASK deletion. **Figure S3.** CASK deletion from neurons does not affect cerebellum.** Figure S4.** Generation of CASK(+/-) heterozygous mutant mice. **Figure S5.** External granular layer of cerebellum is normal during development of CASK(+/-) heterozygous mutant mice. **Figure S6.** Characterization of CASK(+/-) heterozygous mutant mice. **Figure S7.** Schematic diagram depicting cell autonomous and non-cell autonomous reduction in cell numbersas possible cause of microcephaly. **Figure S8.** Table showing the nucleotide sequences of CASK shRNA used in the study. **Figure S9.** CASK knockdown using shRNA 690 reduces cellular respiration and proliferation. (PDF 73672 kb) Additional file 2: Movie 1. Demonstrates the movement and interaction of CASK neuronal knockout mice and wild-type littermate. The smaller mouse is the mutant mouse. (MOV 12948 kb) Additional file 3: Movie 2. Demonstrates recurrent severe seizures in neuronal CASK knockout mice at 21 days after birth. (MOV 69740 kb)
