# Supplementary material for: X-linked intellectual disability gene CASK regulates postnatal brain growth in a non-cell autonomous manner
Source: Acta Neuropathol Commun. 2016 Mar 31;4:30. doi: 10.1186/s40478-016-0295-6 (PMC4818453; doi:10.1186/s40478-016-0295-6)
Supplement: Additional file 1: Figure S1. — Description of mouse lines used and effect of cre expression on floxed CASK gene. Figure S2. CASK deletion from neurons and characterization of female heterozygous mice with neuronspecific CASK deletion. Figure S3. CASK deletion from neurons does not affect cerebellum. Figure S4. Generation of CASK(+/-) heterozygous mutant mice. Figure S5. External granular layer of cerebellum is normal during development of CASK(+/-) heterozygous mutant mice. Figure S6. Characterization of CASK(+/-) heterozygous mutant mice. Figure S7. Schematic diagram depicting cell autonomous and non-cell autonomous reduction in cell numbersas possible cause of microcephaly. Figure S8. Table showing the nucleotide sequences of CASK shRNA used in the study. Figure S9. CASK knockdown using shRNA 690 reduces cellular respiration and proliferation. (PDF 73672 kb) [file 40478_2016_295_MOESM1_ESM.pdf]

Supplementary Materials:

Fig. S1. Description of mouse lines used and effect of cre expression on floxed *CASK* gene

(A) Table describing various mouse lines used in this study **(**B) Immunoblot from cortical cultures with indicated antibody; fl-CASK indicates *CASK* gene is floxed, and fl-CASK; Cre indicates the neuronal culture with floxed *CASK* gene transduced with Cre-recombinase expressing lentivirus. (C) Quantitation of CASK expression relative to liprin-α3 expression. Data is plotted as mean ± SEM, n = 3.

Fig. S2. *CASK* deletion from neurons and characterization of female heterozygous mice with neuron-specific *CASK* deletion

(A) Representative brain images of Rosa-CAG-LSL-tdTomato mice with or without synapsin-Cre; note the ubiquitous expression of tdTomato with synapsin-Cre. (B) Sections from cerebellum and cortex showing Cre-mediated recombination in most neurons. (C) Representative images of brain from mice with indicated genotypes (the mice are shown in lower panel). (D) Shows that mice heterozygous for neuron-specific *CASK* deletion do not exhibit hind-limb clasping phenotype. (E) Comparison of brain weight of mice with indicated genotypes. Bar graphs are plotted as mean ± SEM; n = 4. (F) Comparison of body weight of mice with indicated genotypes.

Fig. S3. *CASK* deletion from neurons does not affect cerebellum

Representative images of H&E stained cerebella from mice of indicated genotype, demonstrating that *CASK* neuronal knockout mice have properly layered cerebella which are indistinguishable from the control mice.

Fig. S4. Generation of *CASK*$^{(+/-)}$ heterozygous mutant mice

Schematic diagram of crosses for generating founder mice for the *CASK*$^{(+/-)}$ colony. Female mice with *CASK* deleted in ova were crossed with the wild-type C57Bl6 males to generate *CASK*$^{(+/-)}$ colony.

Fig. S5. External granular layer of cerebellum is normal during development of *CASK*$^{(+/-)}$ heterozygous mutant mice

(A) Cerebella from the postnatal day 5 mouse pups were dissected and fixed in PFA. Parasaggital sections were stained with a green nucleic acid stain to reveal the external granular layer (EGL). (B) Quantitation of thickness of EGL is plotted as mean ± SEM; n = 3.

Fig. S6. Characterization of *CASK*$^{(+/-)}$ heterozygous mutant mice

(A) Representative images of brain sections depicting corpus callosum (CC) stained with hematoxylin; note that there is no apparent defect. (B) Representative images of neuromuscular junction stained with bungartoxin-alexa488 from the extensor digitorum longus muscles. We did not find any obvious differences in the size, distribution, or number (quantitation not shown).

Fig. S7. Schematic diagram depicting cell autonomous and non-cell autonomous reduction in cell numbersas possible cause of microcephaly

(A) Schematic representation of normocephaly in wild-type mice. Green circles represent cells expressing *CASK* in a *CASK*$^{(+/+)}$ brain. (B) Schematic representation of microcephaly in a non-cell autonomous manner in *CASK*$^{(+/-)}$ brain. Green circles represent cells expressing *CASK* and red circles represent *CASK* deleted cells. A uniform reduction in both cell types will result in ~ 50% reduction in total *CASK* level as measured from whole brain homogenates. (C) Schematic representation of microcephaly in a cell autonomous manner in *CASK*$^{(+/-)}$ brain. Specific loss of only *CASK* deleted cells will result in no significant reduction in total CASK level in whole brain homogenates.

Fig. S8. Table showing the nucleotide sequences of *CASK* shRNA used in the study

Fig. S9. *CASK* knockdown using shRNA 690 reduces cellular respiration and proliferation (A) Representative blot showing *CASK* knockdown in HEK 293 cells transfected with four individual *CASK* shRNAs. (B) Quantitation of bands seen in A, bar graphs are plotted as mean ± SEM ; n = 3. (C) Mitochondrial respiration measurements in *CASK* knockdown HEK 293 cells and wild-type HEK 293 cells. Respiration rate is normalized to total protein levels and data is expressed as percent wild-type. Data is plotted as mean ± SEM. (n = 4; * indicates $p < 0.05$). (E) Hemocytometer cell count in *CASK* knockdown HEK 293 cells (shRNA 690) compared to the wild-type control. Data plotted as mean ± SEM. (N = 4; * indicates $p < 0.05$).

Movie S1: Demonstrates the movement and interaction of *CASK* neuronal knockout mice and wild-type littermate. The smaller mouse is the mutant mouse.

Movie S2: Demonstrates recurrent severe seizures in neuronal *CASK* knockout mice at 21 days after birth.

A

Mouse line	Original reference	Recombination observed in	Original source
CASK^{floxed}	Atasoy et. al. 2007	NA	The Jackson Laboratory
LSLtdTomato	Madisen et. al. 2010	NA	The Jackson Laboratory
ZP3-cre	Lewandoski et. al. 1997	Oocytes	The Jackson Laboratory
Synapsin-cre	Zhu et. al. 2001	Post mitotic neurons, recombination begins as early as E12.5.	The Jackson Laboratory
Math5-cre	Yang et. al. 2003	In many types of postmitotic neurons including granule cells of cerebellum. Recombination begins at E11.5.	The Jackson Laboratory
PCP2-cre	Barski et. al. 2000	Purkinje cells in cerebellum, recombination first observed at postnatal day 6.	The Jackson Laboratory

B **C**

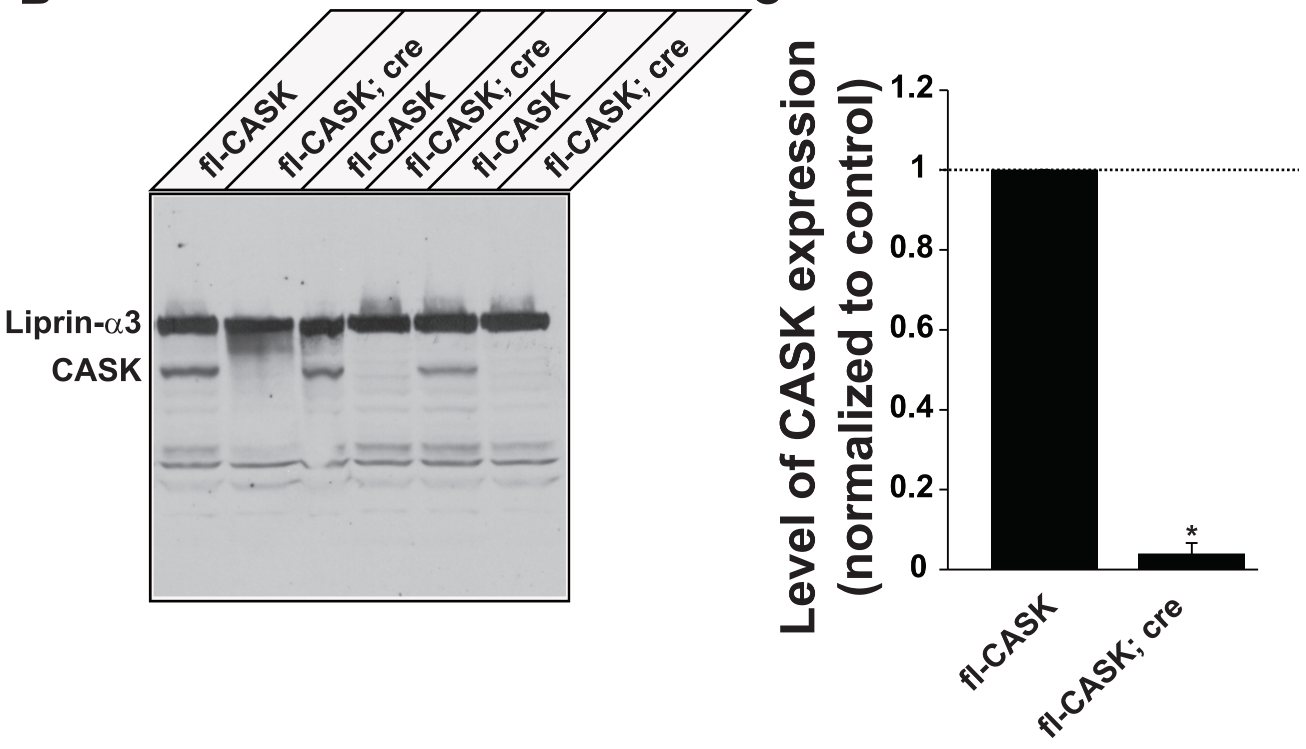

Figure S1

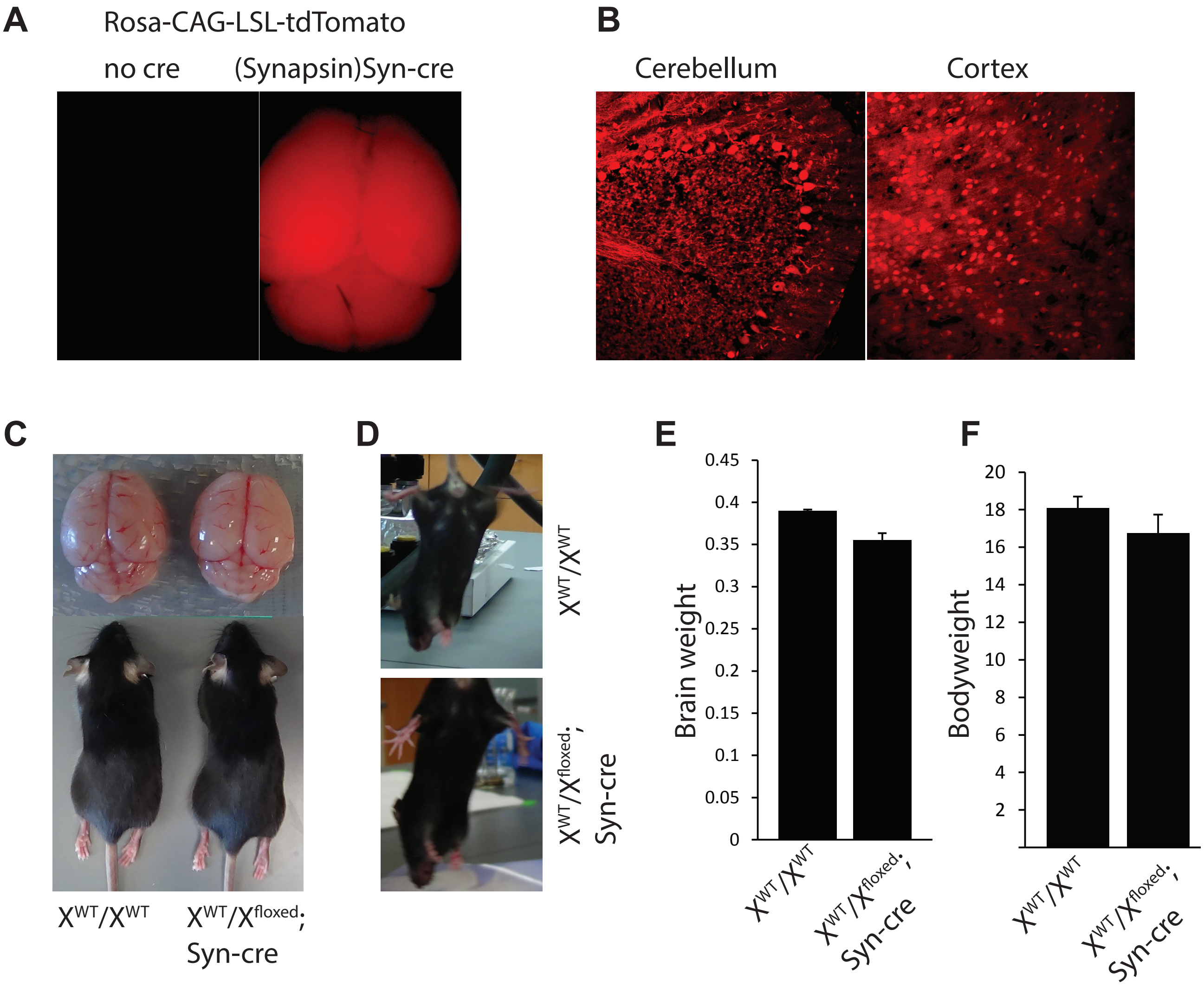
A
Rosa-CAG-LSL-tdTomato
no cre
(Synapsin)Syn-cre
B
Cerebellum
Cortex
C
X^WT/X^WT
X^WT/X^floxed;
Syn-cre
D
X^WT/X^WT
X^WT/X^floxed;
Syn-cre
E
Brain weight
0.45
0.4
0.35
0.3
0.25
0.2
0.15
0.1
0.05
0
X^WT/X^WT
X^WT/X^floxed;
Syn-cre
F
Bodyweight
20
18
16
14
12
10
8
6
4
2
X^WT/X^WT
X^WT/X^floxed;
Syn-cre

Figure S2

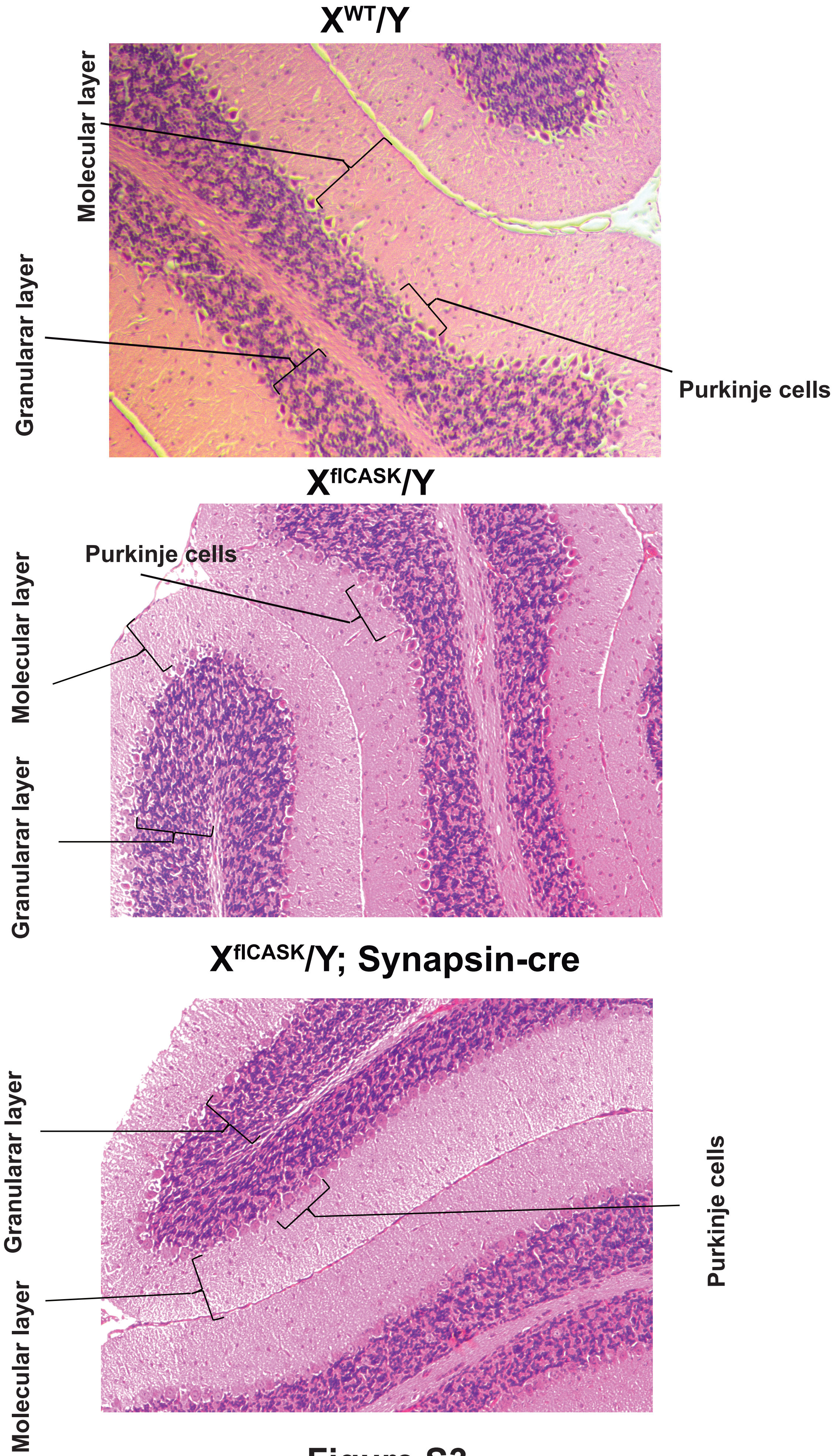
X^{WT}/Y
Molecular layer
Granularar layer
Purkinje cells
X^{flCASK}/Y
Purkinje cells
Molecular layer
Granularar layer
X^{flCASK}/Y; Synapsin-cre
Granularar layer
Purkinje cells
Molecular layer

Figure S3

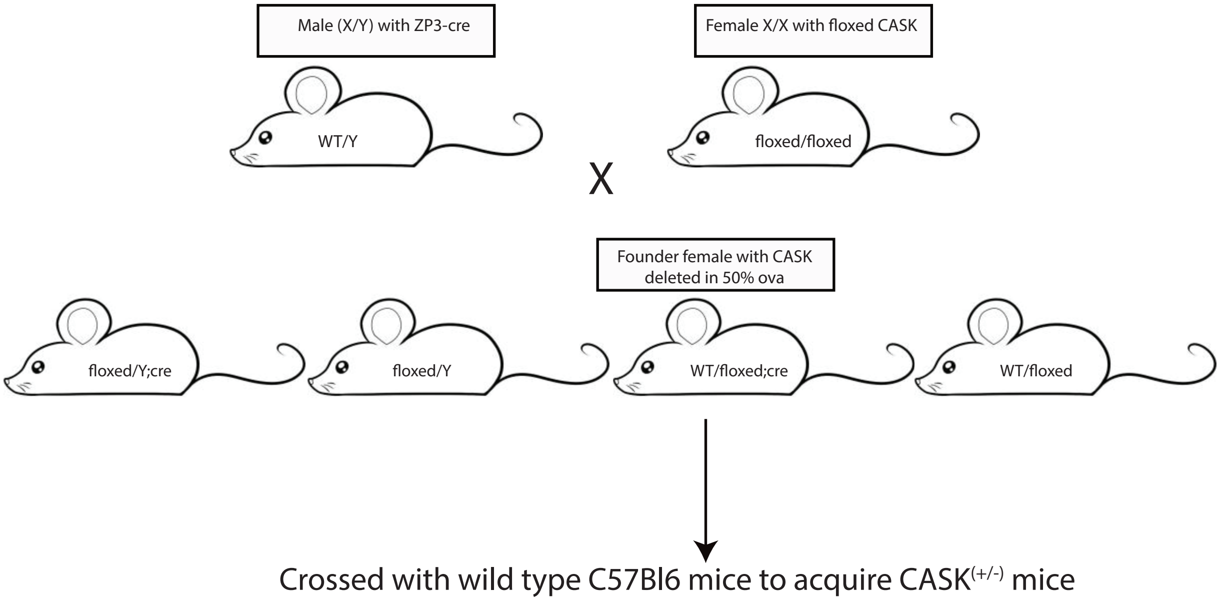
Male (X/Y) with ZP3-cre
Female X/X with floxed CASK
WT/Y
floxed/floxed
X
Founder female with CASK deleted in 50% ova
floxed/Y;cre
floxed/Y
WT/floxed;cre
WT/floxed
Crossed with wild type C57Bl6 mice to acquire CASK(+/-) mice

Figure S4

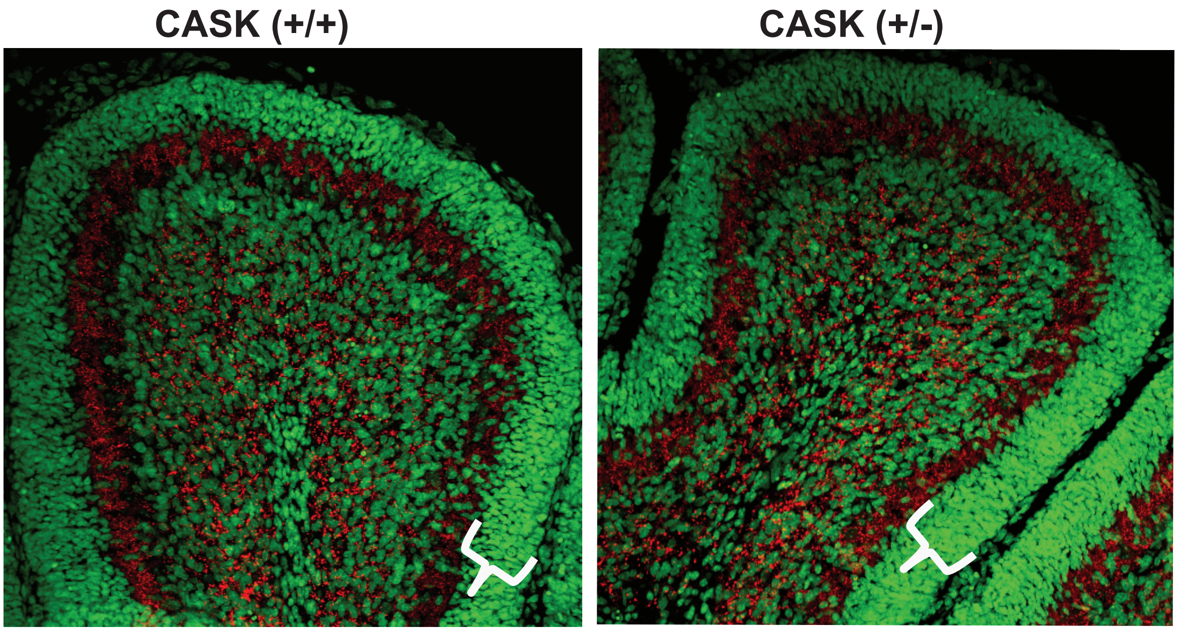

Postnatal day 5 cerebellum sections

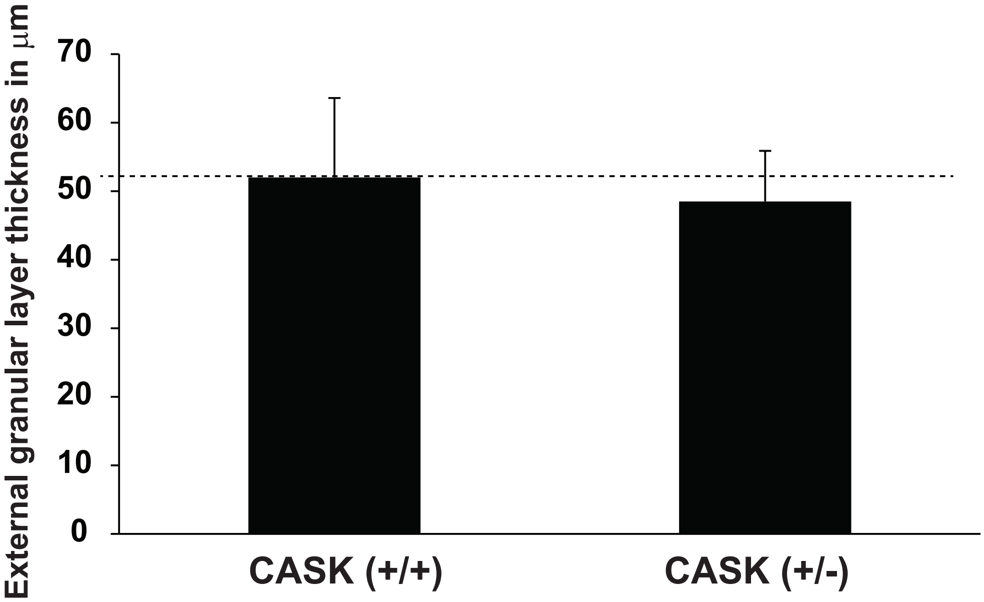

Figure S5

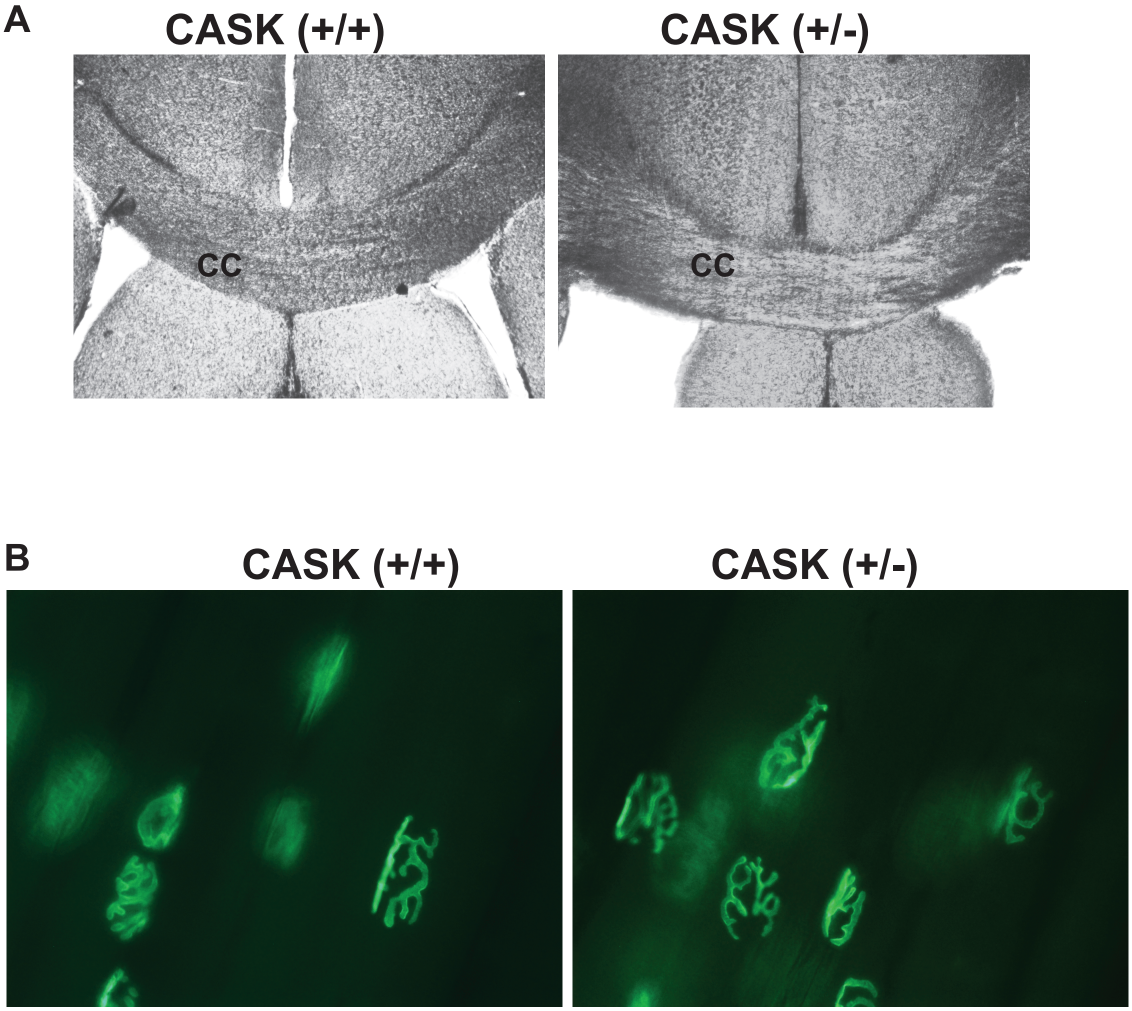

Figure S6

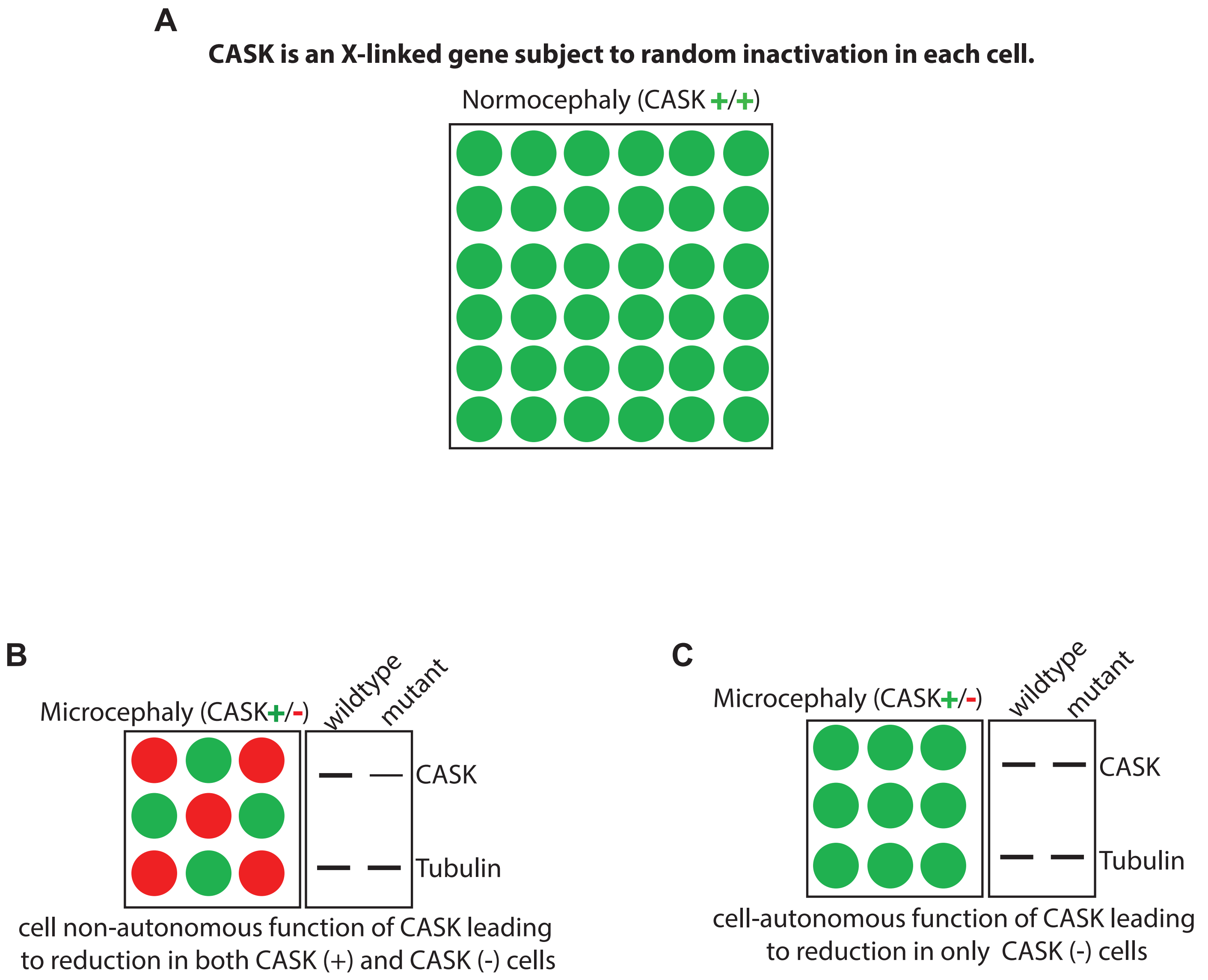
A
CASK is an X-linked gene subject to random inactivation in each cell.
Normocephaly (CASK +/+)
B
Microcephaly (CASK+/-)
wildtype
mutant
CASK
Tubulin
cell non-autonomous function of CASK leading to reduction in both CASK (+) and CASK (-) cells
C
Microcephaly (CASK+/-)
wildtype
mutant
CASK
Tubulin
cell-autonomous function of CASK leading to reduction in only CASK (-) cells

Figure S7

shRNA	Mature antisense
Sh690	TGCTCCCAGTTATGCTCAGAT
Sh691	AATGCCAGCTTCTTTACAGGG
Sh692	TTTGCGTCGTTATTCTCAGGG
Sh693	TTTGGTTGGGTAGTTGATGGC
Sh694	AATACGCTTTAGTTCCTTTGC

Figure S8

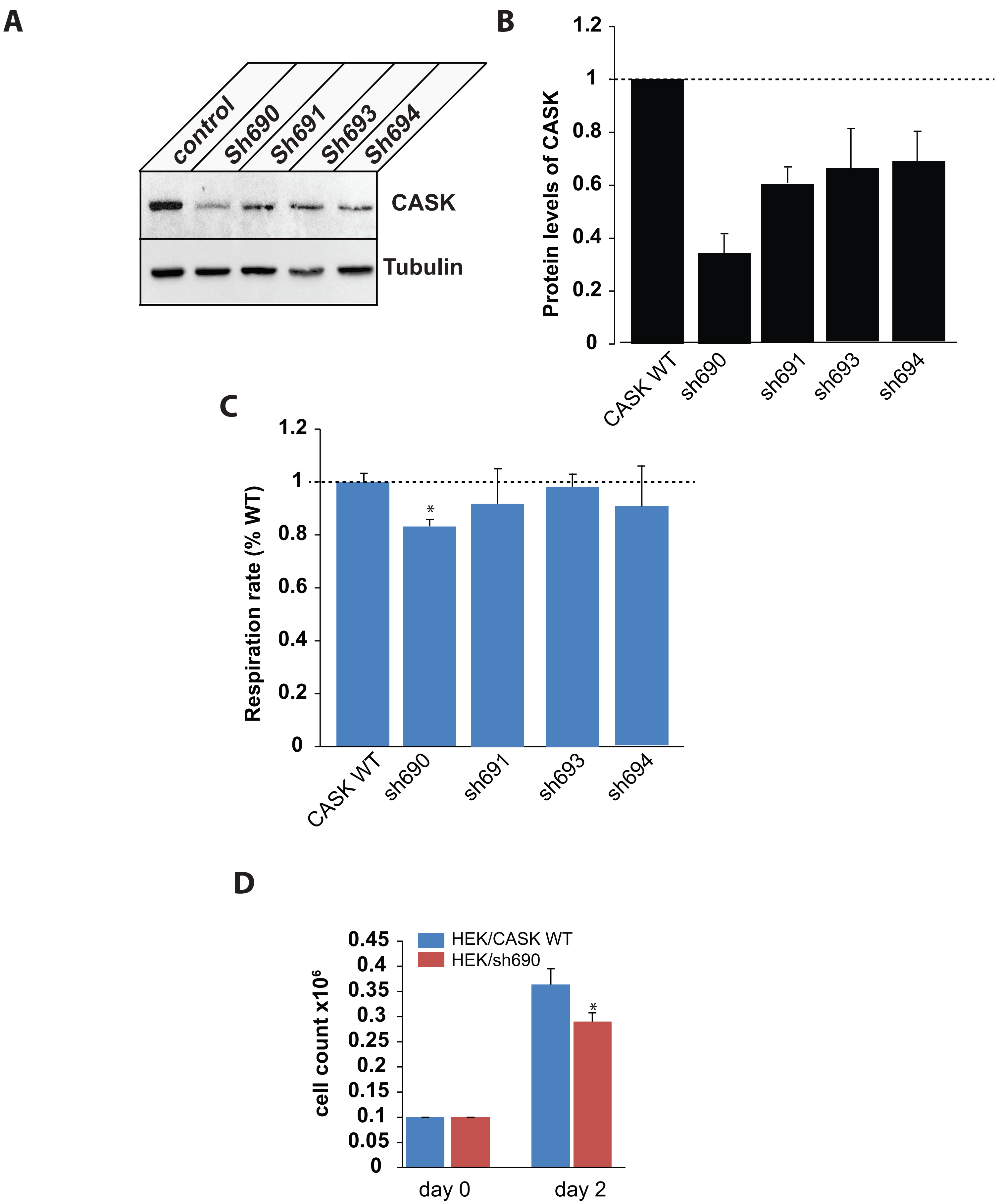
A
control
Sh690
Sh691
Sh693
Sh694
CASK
Tubulin
B
Protein levels of CASK
1.2
1
0.8
0.6
0.4
0.2
0
CASK WT
sh690
sh691
sh693
sh694
C
Respiration rate (% WT)
1.2
1
0.8
0.6
0.4
0.2
0
*
CASK WT
sh690
sh691
sh693
sh694
D
cell count x10⁶
0.45
0.4
0.35
0.3
0.25
0.2
0.15
0.1
0.05
0
HEK/CASK WT
HEK/sh690
*
day 0
day 2

Figure S9
